# Neurofeedback and Attention-Deficit/Hyperactivity-Disorder (ADHD) in Children: Rating the Evidence and Proposed Guidelines

**DOI:** 10.1007/s10484-020-09455-2

**Published:** 2020-03-23

**Authors:** Martijn Arns, C. Richard Clark, Mark Trullinger, Roger deBeus, Martha Mack, Michelle Aniftos

**Affiliations:** 1 Research Institute Brainclinics, Brainclinics Foundation, Bijleveldsingel 34, 6524 AD Nijmegen, The Netherlands; 2 Department of Experimental Psychology, Utrecht University, Utrecht, The Netherlands; 3 neuroCare Group, Nijmegen, The Netherlands; 4 Applied Neuroscience Society of Australasia (ANSA), Brisbane, Australia; 5 Mylne Street Mental Health (MSMH), Toowoomba City, Australia; 6 Biofeedback Certification International Alliance – Australia (BCIA-A), Adelaide, Australia; 7 College of Education, Psychology and Social Work, Flinders University of South Australia, Adelaide, SA Australia; 8 The Chicago School of Professional Psychology, Washington, D.C. USA; 9 NeuroThrive, LLC, Lutherville, MD USA; 10 Department Psychology, University of North Carolina at Asheville, Asheville, NC USA; 11 International Society of Neurofeedback and Research, Miami, FL USA; 12 neuroCare Group Melbourne, Melbourne, Australia; 13 APS Neurofeedback Interest Group, Melbourne, Australia

**Keywords:** ADHD, Neurofeedback, Review, Remission, Effect size

## Abstract

Stimulant medication and behaviour therapy are the most often applied and accepted treatments for Attention-Deficit/Hyperactivity-Disorder (ADHD). Here we explore where the non-pharmacological clinical intervention known as neurofeedback (NFB), fits on the continuum of empirically supported treatments, using standard protocols. In this quantitative review we utilized an updated and stricter version of the APA guidelines for rating ‘well-established’ treatments and focused on efficacy and effectiveness using effect-sizes (ES) and remission, with a focus on long-term effects. Efficacy and effectiveness are compared to medication and behaviour therapy using benchmark studies. Only recent systematic reviews and meta-analyses as well as multi-centre randomized controlled trials (RCT’s) will be included. Two meta-analyses confirmed significant efficacy of standard neurofeedback protocols for parent and teacher rated symptoms with a medium effect size, and sustained effects after 6–12 months. Four multicenter RCT’s demonstrated significant superiority to semi-active control groups, with medium-large effect sizes end of treatment or follow-up and remission rates of 32–47%. Effectiveness in open-label studies was confirmed, no signs of publication bias were found and no significant neurofeedback-specific side effects have been reported. Standard neurofeedback protocols in the treatment of ADHD can be concluded to be a well-established treatment with medium to large effect sizes and 32–47% remission rates and sustained effects as assessed after 6–12 months.

## Introduction

Currently, stimulant medication and behaviour therapy are the most often applied and accepted treatments for Attention-Deficit/Hyperactivity-Disorder or ADHD. In the acute treatment of ADHD, these treatments have both large effects and considerable remission rates (Cortese et al. 2018; Swanson et al. 2001). This paper explores where the non-pharmacological clinical intervention known as EEG (electroencephalogram) biofeedback, otherwise known as Neurofeedback (NFB), fits on the continuum of empirically supported treatments.

## Rating Clinical Efficacy: Empirically Supported Treatments

In 1993, the first criteria for rating ‘Empirically Validated Psychological Treatments’ were published by the American Psychological Association (APA). These treatments were later described as ‘Empirically Supported Treatments’ (Chambless and Hollon 1998). This framework for establishing treatment guidelines, has heavily influenced APA’s view on Evidence Based Practice in Psychology (APA Presidential Task Force on Evidence-Based Practice 2006), as reviewed by Hollon et al. (2014). These original criteria have also been applied to neurofeedback and biofeedback applications, as proposed in 2002 by the AAPB and SNR Efficacy Task Force in this journal (La Vaque et al. 2002) and applied to neurofeedback in ADHD by Monastra et al. (2005) and later by Arns et al. (2009,2014).

The APA criteria developed for establishing treatment guidelines is characterised by two constructs: (1) *Treatment efficacy* the systematic and scientific evaluation of whether a treatment works, with efficacy graded into various levels, with ‘*efficacious and specific*’ representing the highest level of efficacy, and (2) *Effectiveness* (also termed ‘Clinical Utility’): the applicability, feasibility, and usefulness of the intervention. This construct is designed to assess the generalizability of the intervention into everyday clinical practice (American Psychological Association 2002; Chambless 1993; Chambless and Hollon 1998).

These APA criteria have now been around for more than a quarter-century and several updates have been proposed in response to criticisms that some of the criteria are outdated (Southam-Gerow and Prinstein 2014; Tolin et al. 2015). The first main criticism is that the APA criterion for two independent studies to demonstrate efficacy may not account for mixed findings or publication bias. To address this, it has been recommended that there is instead a reliance on recent systematic reviews and meta-analyses consisting of more than two independent RCT’s. A second criticism is that use of measures of symptom reduction as an outcome might be too limited and that measures of clinical relevance such as effect size (ES) and remission rate should be factored in. Thus, the current paper strengthens the APA criteria by accommodating these more rigorous recommendations, and in this way adopts stricter guidelines for assessing the evidence base for neurofeedback treatment, as summarized in Table 1.

**Table 1:** Comparison of the original APA guidelines for identification of ‘well-established’ treatment studies, as described in (Chambless and Hollon 1998) (Left) with stricter criteria proposed for neurofeedback, including recommendations by Tolin et al. 2015 and others (Right). The stricter criteria were used in the present quantitative review for selection of ADHD treatment studies

Original APA guidelines	Stricter guidelines used in this review
I: At least two good between- group design experiments demonstrating efficacy in one or more of the following ways:	I: Efficacy
A. Superior (based on statistical significance alone) to pill or psychological placebo or to another treatment	A. For rating efficacy rely on a. Systematic review and meta-analysis (recent: last 2 years) b. Multi-centre Randomized Controlled Trials (RCTs)
B. Equivalent to an already established treatment in experiments with adequate statistical power, considered to be approximately 30 per group	B. Consider clinical significance (Remission) in addition to statistical significance (Cohen’s D)
	C. Consider long-term efficacy in addition to short-term efficacy
	D. Statistical superiority to semi-active control groups or inert placebo
	E. Equivalence to already established treatment (active treatments)
	F. Consider bias via meta-analysis, e.g. publication bias
*OR*	*AND*
II: A large series of single-case design experiments (N > 9) demonstrating efficacy. These experiments must have:	II: Effectiveness
A. Used good experimental designs and	A. Address generalization of research findings to non-research settings and diverse populations: Open-label studies
B. Compared the intervention to another treatment as in I A	B. Cohen’s D and Remission rates
	C. Safety and Side-effect profile
	D. Cost–benefit analysis
*Further criteria for both I and II*	
III: Experiments must be conducted with treatment manuals	III: Experiments must be conducted with treatment manuals. In relation to neurofeedback studies, this is operationalized to restrict selection to those studies employing standard protocols: TBR, SMR and SCP protocols (see text for detail)
IV: Characteristics of the client samples must be clearly specified	IV: Characteristics of the client samples must be clearly specified
V: Effects must have been demonstrated by at least two different investigators or investigating teams	V: Independent replication

Therefore, the primary purpose of this quantitative review is to evaluate the efficacy and effectiveness of neurofeedback intervention. This review will not examine in any detail the *mechanisms of change* resulting from neurofeedback, which have been reviewed elsewhere (e.g. Sitaram et al. 2016). That said, efficacy and effectiveness of neurofeedback are unlikely to be mediated by any single mechanism or intervention protocol and multiple mechanisms may contribute. These include primary reinforcement of targeted neurophysiological activity via operant conditioning, secondary reinforcement due to the psychological factors implicit in treatment protocols and, in some conditions, synergistic gains when the method is conjoined with other treatments (e.g. psychological therapy, coaching, sleep hygiene etc.). Hence, when we speak of neurofeedback, we have to be aware of this possible *multifactorial* aspect. Analogously, such multifactorial impacts are also seen in pharmacotherapy, with the efficacy of most drugs mediated by action on more than one neurotransmitter system (Sanchez et al. 2014), but also the colour and shape of drugs (de Craen et al. 1996), and a similar multifactorial view is known for psychotherapy.

## What is Neurofeedback? A Brief History

Neurofeedback is a therapeutic technique that seeks to modulate and retrain brain function to address neurological and/or psychological symptoms of concern. One of the original demonstrations of the potency of neurofeedback involved what is termed the Sensori-Motor Rhythm (SMR), an EEG rhythm in the low beta range (12–15 Hz) derived from the EEG from the region of the scalp located over the sensori-motor strip. Sterman and Friar (1972) demonstrated the first anticonvulsant effects in epilepsy. Lubar and Shouse (1976) then described the successful application of this technique in a child with hyperkinetic syndrome, a condition closely resembling what is now termed ADHD. Several years later, these findings were replicated in a larger study (Shouse and Lubar 1979) and extended to the use of a modified protocol involving not only training of SMR but also of a slower rhythm called theta (4–7 Hz). This revised protocol was named Theta/Beta Neurofeedback (Lubar and Lubar 1984). Subsequently, a slightly different form of neurofeedback was described, called Slow Cortical Potential (SCP) neurofeedback that was shown to not only have anticonvulsive properties in epilepsy (Rockstroh et al. 1993) but also clinical effects in ADHD (Heinrich et al. 2004). SCP's are DC shifts related to positive or negative shifts in broad sheets of glial cells, representing increased activation (negativity) or decreased activation (positivity) and these very slow oscillations in the EEG associated with readiness that transfer into daily life during learning. Not all EEG frequencies being trained have been shown to be efficacious in these conditions. For example, training of the posterior alpha rhythm (8–13 Hz) has failed to show clinical benefit in either hyperkinetic syndrome (Nall 1973) and epilepsy (Rockstroh et al. 1993), suggesting *specificity* in the EEG parameter trained for successful neurofeedback. Therefore, the first three well-investigated protocols (SMR, TBR and SCP) have also been termed ‘standard neurofeedback protocols’. For a more detailed overview of the history of neurofeedback and these ‘standard neurofeedback protocols’ see Arns et al. (2014).

In this review we will limit evaluated studies to neurofeedback studies that have employed ‘standard neurofeedback protocols’ as outlined in the previous section. This entails the exclusion of some double-blind placebo-controlled studies such as Arnold et al. (2013) and Van Dongen-Boomsma et al. (2013), since both used neurofeedback protocols and approaches that have not been studied before, and thus no valid conclusions can be drawn from these studies as to whether the effects (or lack thereof) are the result of these non-standard protocols or can be attributed to neurofeedback in a broader sense. Also, the question of whether neurofeedback can and should be evaluated with double-blind placebo-controlled designs is a complex topic, as discussed in detail elsewhere (Arns et al. 2014; Sorger et al. 2019), and is beyond the scope of this review. Of relevance here, is that by using the APA framework, neurofeedback is evaluated in the same way as psychological treatments, and in this regard, it is notable that for most effective psychological treatments, double-blind placebo-controlled studies are not possible and have not been conducted. Furthermore, by placing the effects and remission rates in the broader landscape of ADHD treatments, the reader will be provided with the relative merits of a range of treatment modalities.

## Parent and/or Teacher Assessment of ADHD for the Rating of Clinical Efficacy?

A recent development initiated by the European ADHD Guidelines Group (EAGG; Sonuga-Barke et al. 2013), for rating efficacy of non-pharmacological and pharmacological treatments, has strongly influenced the evaluation of neurofeedback and other treatments in the field of ADHD in relation to rater-eligibility. In the approach advocated, meta-analyses are conducted on two levels—the first based on the ‘most-proximal raters’, (i.e. those closest to the treatment setting, most often parents) and the second based on ‘probably-blinded raters’ (i.e. those most distal to the treatment setting, e.g. teachers). This approach has been applied in several meta-analyses to date, focusing respectively on non-pharmacological (Sonuga-Barke et al. 2013), neurofeedback (Cortese et al. 2016) and pharmacological (Cortese et al. 2018) approaches. However, note that parent and teacher ratings have been equally often used in methylphenidate clinical trials (Faraone and Buitelaar 2010), clarifying that both parent or teacher ratings have been considered valid treatment endpoints to rate clinical efficacy.

There are, however, grounds on which to question the reliability of teacher ratings for rating clinical efficacy (see: Minder et al. 2018). For example, up to the age of 12, children usually have a single teacher that can generally be assumed to have a relatively extended knowledge of the child’s behaviour. Beyond that age, teacher ratings become less reliable, as more teachers become involved in the child’s education and for shorter periods of time. Further, with teachers tending to change annually with children over the age of 12, there is less scope for within-teacher assessment for reliable follow-up studies. Finally, it can be argued that teachers may be more sensitive in their ratings to psychopharmacological interventions that exert their effects quickly (e.g. methylphenidate and modafinil) relative to treatments with a slower onset of effect (e.g. behavioural interventions, atomoxetine, bupropion and guanfacine). This was actually confirmed by a recent EAGG meta-analysis, where medium to large effect sizes based on teacher ratings were found for interventions involving methylphenidate and modafinil, but only small-medium, non-significant effect sizes found for atomoxetine, bupropion and guanfacine (Cortese et al. 2018). Further arguments and data regarding poor reliability of teacher ratings is outlined in Minder et al. (2018), who elaborate on discrepancies between parent and teacher rated behaviour and the propensity for bias in teacher rated assessment. As a result, we will focus primarily on parent rated symptoms in this study due to the focus on long-term effects and will do so systematically for all treatment modalities in order to validly compare efficacy *across* treatments.

## Methods

### Study Selection

The selection of studies was undertaken using an adaptation of the original APA guidelines (Chambless and Hollon 1998), as visualized in Table 1 on the right. The two most recent systematic reviews were identified resulting in the Van Doren meta-analysis (Van Doren et al. 2018) that focused on long-term outcomes of neurofeedback in ADHD and the ADHD Guidelines Group meta-analysis (Cortese et al. 2016) that focused on acute effects of neurofeedback in ADHD. The studies that were recovered in the systematic searches of those reviews were evaluated on the following criteria (also in line with criteria as per Table 1): (1) Multicentre Randomized Controlled Trial; (2) Primary diagnosis of ADHD; (3) Use of standard protocols (SMR, TBR and/or SCP protocols); and (4) mean child age < 18 years old. Conclusions from these meta-analyses, as well as from the individual studies that met criteria, are provided in the results.

In order to provide a frame of reference, and to compare neurofeedback to other treatments, identical outcome measures were extracted from (1) the multi-centre NIMH Multimodal Treatment Study of Children with ADHD (MTA) (The MTA Cooperative Group 1999), that is regarded a benchmark for ADHD treatments. In the MTA study, 579 children were randomized into four arms: multicomponent behaviour therapy (BEH), medication (MED), combined treatment (COMB) and routine community care (CC). It should be noted that remission rates for the MTA were based on inattention, hyperactivity, impulsivity *and* ODD symptoms. (2) The International Study to Predict Optimized Treatment in ADHD (iSPOT-A) study (Arns et al. 2018; Elliott et al. 2017), which is a large non-industry sponsored multi-centre open-label, treatment as usual (TAU) trial of methylphenidate treatment in ADHD. This study was conducted in the Netherlands, Australia and the US, involving a sample of 336 children and adolescents with ADHD. For further details also see Arns et al. (2018) and Elliott et al. (2017).

The MTA results were used to assess and compare *efficacy* across treatment modalities (A). The iSPOT-A results were used to assess and compare *effectiveness* across treatment modalities (B).

### Outcome Measures and Analysis Procedures

The outcome measures adopted to assess clinical efficacy of neurofeedback in ADHD are described below. They are described relative to the constructs of clinical efficacy recommended from APA criteria: (A) Treatment Efficacy and (B) Effectiveness, following the recent recommendations of Tolin et al. (2015), as described earlier.

(A) Treatment Efficacy: Here, three sets of outcome measures are adopted:
  1. Acute Pre-post treatment effect size (ES): This metric reflects the improvement of the group from pre-treatment to post-treatment in terms of standard deviations (SD) and expressed as Cohen’s D, as commonly used in meta-analyses for parent rated total symptoms (using the scale that was defined primary endpoint in a given study). For meta-analyses both within-group and between-group ES will be reported and discussed. Conventionally, an ES > 0.3 is considered a small clinical effect, an ES > 0.5 considered a medium sized effect and an ES > 0.8 considered a large clinical effect. Most medication focused meta-analyses report ES as a *between group* measure (medication vs. placebo); however, given the variety of control groups employed in neurofeedback studies (e.g. active controls including medication as well as semi-active controls such as attention training) we focus here on within-group ES, an approach that also allows us to compare directly the efficacy of neurofeedback to that of behavioural treatments and medication (also see: Arns et al. (2009), Van Doren et al. (2018) for more detail).
  2. Long-term Pre-Follow-up treatment ES: The same as above, but also contrasting the pre-treatment and follow-up treatment means and SD’s.
  3. Remission: Remission (i.e. loss of diagnostic status) is defined as an ADHD rating scale item mean of ≤ 1 (Steele et al. 2006; Swanson et al. 2001), published for the MTA trial (Swanson et al. 2001). When remission was not published, the authors were contacted (up to 2 times) to request remission rates. Such a request was fulfilled by (Arns et al. 2012; 2018; Gevensleben et al. 2009; Strehl et al. 2017), could not be fulfilled by (Kropotov et al. 2005; Monastra et al. 2002; Steiner et al. 2014) for various reasons and no response (Geladé et al. 2016).
(B) Effectiveness: Effectiveness is operationalised here to refer to the generalizability of neurofeedback efficacy in the treatment of ADHD to everyday clinical practice, when standardised practices are more difficult to obtain. We use the measures of Effect Size (ES) and Remission described under Treatment Efficacy but then applied to open-label studies.

## Results

The primary RCT’s and Open Label trials included in this analysis for assessment of the clinical efficacy and effectiveness of neurofeedback are listed in Table 2.

**Table 2:** Studies included in this quantitative review

Source	N_(Act-Ctrl)_	Active Treatment	Control
(1) Strehl et al. (2017)	73–67	SCP	EMG Biofeedback
(2) Gevensleben et al. (2009, 2010)	59–35	SCP and TBR	Cognitive training
(3) Geladé et al. (2016, 2018)	39–37–36	TBR	Exercise and MPH
(4) Steiner et al. (2014)	34–32–36	SMR	Cognitive training and Waitlist
NIMH-MTA Study: MTA (1999)			
(5) MTA Combined	145	MPH & Behavioral	
(6) MTA Medication	144	MPH	
(7) MTA Behavioral	144	Behavioral	
(8) MTA Community Care	146	Community Care	
(9) Arns et al. (2012)	21	SMR, TBR or SCP	QEEG-*informed*
(10) Monastra et al. (2002)	51	TBR	TBR-preselection
(11) Kropotov et al. (2005)	86	SMR, TBR	Open Label
(12) iSPOT-A: Arns et al. (2018)	336	MPH	Open Label

*NFB* Neurofeedback, *MPH* Methylphenidate, *SMR* Sensori-Motor Rhythm protocol, *TBR* Theta/Beta Ratio protocol, *SCP* Slow Cortical Potential protocol, *Act* Active treatment, *Ctrl* control condition. Studies 1–8 are RCT’s and studies 9–12 are Open-Label effectiveness studies

### Systematic Reviews and Meta-analyses

Two systematic reviews and meta-analyses were identified: one that focused on long-term outcomes of neurofeedback in ADHD (Van Doren et al. 2018) and another, the EAGG meta-analysis that focused on acute outcomes of neurofeedback (Cortese et al. 2016). For standard protocols, Cortese et al. (2016) found an overall significant Standard Mean Difference (SMD, comparable to a Cohen’s D) for Total ADHD symptoms (ES = 0.45, parent rated; ES = 0.36, teacher rated) and Van Doren and colleagues (Van Doren et al. 2018) found between-group ES for inattention of 0.52 that increased to 0.57 at follow-up. Within-group ES for van Doren was 0.65 that increased to 0.83 at follow-up.

Regarding publication bias the fail-safe statistic was > 100 studies (Van Doren et al. 2018). Based on funnel plots and Egger test results (Cortese et al. 2016), no signs of publication bias were deemed to be present.

### Treatment Efficacy

**Fig. 1:**
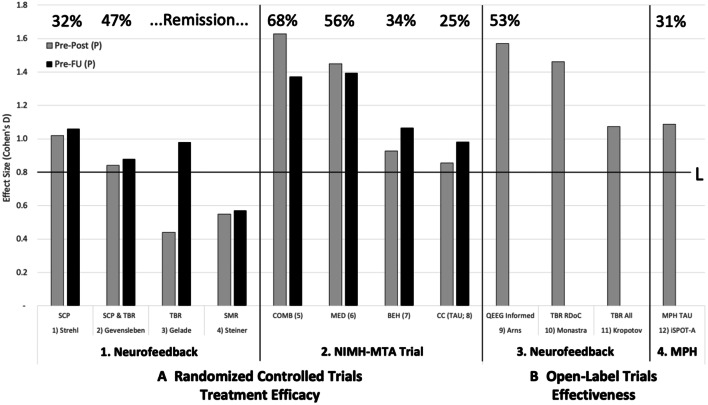
The landscape of ADHD treatments with pre-post treatment effect sizes (Cohen’s D; ES) for parent rated overall ADHD symptom improvement (grey, Pre-Post) and from pre-treatment to follow-up (black, Pre-FU), with indicators illustrating large ES (L: D > 0.8) and remission rates listed on top (top line; no data on remission rates was available for studies (3), (4) (10) and (11)). On the left the results for efficacy are depicted and on the right for effectiveness, separated in 1. Neurofeedback RCT’s, 2. NIMH-MTA treatment arms (Combined treatment (COMB), Medication only (MED), Multicomponent Behaviour Therapy (BEH) and Community Care—treatment as usual (CC (TAU)), 3. Neurofeedback open label trials and 4. Methylphenidate (MPH) open label data from the iSPOT-A study. Note the consistent increases in ES for neurofeedback studies from pre-treatment to follow-up, with the opposite trend for the MTA medication arms, indicating a strengthening of effects for neurofeedback over time, without additional treatment. The open label trials also demonstrate the benefit obtained in clinical settings is overall similar or better, relative to clinical trials, whereas the effects of open-label MPH tend to be lower relative to the MTA results. This demonstrates results of neurofeedback translate well into clinical practice (‘effectiveness’ based on standard protocols)

From these systematic reviews, four multicentre RCTs were identified that used standard neurofeedback protocols, as summarized in Table 2. Overall, all studies showed significant benefit for neurofeedback relative to semi-active control groups at post-treatment or at follow-up. In addition, the Geladé study showed no significant difference between the MPH and neurofeedback groups at follow-up (Geladé et al. 2018), although the sample size was not powered sufficiently to claim equivalence. The results are summarised in Fig. 1 (Section A1, Fig. 1). Large (D > 0.8) or medium (D > 0.5) pre-post effect sizes are evident post-treatment and follow-up for three of the four studies and remission rates were 32–47%. The fourth study also produced a positive result, measuring just below the medium ES boundary (d < 0.5). This study by Steiner and colleagues (Steiner et al. 2014) was conducted in a school rather than a psychological clinical setting, a difference that may explain the relative smaller effect size compared to other studies. Of note, is the consistent *increase* in effect size in all four studies from pre-treatment to follow-up, indicating a strengthening in effect without additional treatment for neurofeedback over time.

The results of the MTA study are shown in section A2 of Fig. 1. All modalities examined in this study (Medication, Behaviour, Combined and Community Care) showed a large ES immediately after treatment and at follow up, with remission rates ranging between 25 and 68% but highest for the medication arms (68% COMB and 56% for MED). The efficacy of neurofeedback tended to be comparable to the Behavioural group of the MTA trial, both in ES as well as remission rate.

### Effectiveness

The open label studies identified in the literature were drawn from Arns et al. (2012), Monastra et al. (2002) and Kropotov et al. (2005), see Table 2 for details. All three studies were mainly based on ‘standard protocols’. In the Arns study (Arns et al. 2012) a QEEG-*informed* procedure was used to select the right standard protocol and in the Monastra study (Monastra et al. 2002), subjects were pre-selected on high TBR; therefore, the relatively larger ES in these studies are potentially explained by these individualizations. The results of the Open Label studies of Neurofeedback are shown in section B3 of Fig. 1, and all studies showed large effect sizes following treatment with 53% remission. No follow-up information was available for any of the open label studies. These results indicate that clinical benefit of neurofeedback achieved in clinical practice are equal to, or better than that achieved in RCT’s. Furthermore, these effects compared favourable to the medication arms of the MTA trial as well as the open-label data from the iSPOT-A study.

### Safety

No significant neurofeedback-specific major adverse events have been reported in any study (Strehl et al. 2017; Steiner et al. 2014; Arnold et al. 2013; Lansbergen et al. 2011).

## Discussion

This study assessed the treatment efficacy and effectiveness in ADHD for medication, behavioural and neurofeedback treatments. The efficacy and effectiveness of neurofeedback as a treatment for ADHD, was rated using a stricter version of the APA guidelines to rate well-established treatments (see Table 1).

To summarize the results (also see Fig. 1):

- Based on two independent and recent systematic reviews and meta-analyses, significant efficacy of standard neurofeedback protocols was confirmed for both parent and teacher rated symptoms (Cortese et al. 2016), with a small-medium between-group effect size. Between-group analysis resulted in small-medium ES (Cortese et al. 2016; Van Doren et al. 2018), whereas within-group analysis resulted in large ES and effects were sustained at 6–12 months follow-up (Van Doren et al. 2018).
- Four multicenter RCT’s, employing standard neurofeedback protocols, ranging from EMG biofeedback to attention training, demonstrated significant superiority to semi-active control groups, with medium-large pre-post effect sizes end of treatment or follow-up and remission rates of 32–47%.
- Three open-label neurofeedback studies demonstrate similar or better efficacy compared to the multicenter RCT’s, demonstrating the effects of neurofeedback translate well into clinical practice.
- No signs of publication bias have been found and no significant neurofeedback-specific side effects have been reported in any prior study (Strehl et al. 2017; Steiner et al. 2014; Arnold et al. 2013; Lansbergen et al. 2011).

Therefore, standard neurofeedback protocols in the treatment of ADHD can be concluded to be a well-established treatment, or ‘efficacious and specific’ in line with the APA guidelines.

Comparing clinical benefit observed with neurofeedback, shows clinical efficacy to be comparable to the multicomponent-behaviour therapy arm of the MTA and only marginally below the MTA medication arms. For effectiveness, the results of neurofeedback seem to be more comparable to medication when compared to the results from the open label iSPOT-A study and to the MTA-medication arms. Interestingly, for medication, the clinical benefit as reported for the MTA-medication arms do not seem to generalize to clinical practice. The results for iSPOT-A indicated a 31% remission rate compared to 56–68% for MTA-medication arms and a 33% lower effect size. Even the effect size for the medication arm of the Geladé study was 44% lower compared to the MTA-medication arms, though they did use the NIMH-MTA medication algorithm (Geladé et al. 2018). Therefore, further studies should evaluate how well the effects of the MTA-algorithm translate to clinical practice, i.e. the effectiveness of the MTA-algorithm.

Thus far, most treatment studies have reported effects in terms of effect size or response (often differently defined as 25%, 35%, 50% improvement etc.), and only few studies have used remission as an outcome (see: (Steele et al. 2006; Swanson et al. 2001). Here we opted to define remission using the operationalization from Swanson (Swanson et al. 2001) and due to the clear interpretation of remission (‘no longer meeting diagnostic status’) and clinical relevance, we would recommend the use of remission in more future clinical studies. The remission rates seen across studies between 32 and 68% for controlled treatments, reflect clinically meaningful remission rates, with the lowest rate for the community care arm of the MTA (25%). Remission rates were clearly highest for the MTA medication arms (56–68%), albeit with decreasing effect sizes at follow-up whilst medication use was continued. The remission rates for neurofeedback (32–47%) in this context are reassuring, and there is the clear finding that effects of neurofeedback are sustained without further treatment. A further advantage of neurofeedback is its safety and non-invasiveness.

Several limitations are noted: While the between group meta-analyses were significant, the between-group effect sizes are generally much smaller than the within-group effect sizes. While this could reflect the choice of ‘semi-active’ control groups as a comparator, it also indicates that non-specific effects can play a major role in the overall neurofeedback efficacy. Future studies need to focus more on the exact mechanisms involved in neurofeedback treatment and further disentangle the various influences. Furthermore, neurofeedback should best be seen as an inherently, multifactorial treatment intervention, with potential active factors including primary reinforcement of targeted neurophysiological activity via operant conditioning, secondary reinforcement due to the psychological factors implicit in treatment protocols and, in some conditions, synergistic gains when the method is conjoined with other treatments (e.g. psychological therapy, psycho-education, sleep advice). The study by Steiner et al. (2014), while showing significant benefit compared to a cognitive training as well as a waitlist control group, showed relatively the smallest effect sizes, compared to the three other studies. The fact that this study was conducted in a school setting rather than in a psychological clinical setting, may explain the relative smaller effect size compared to other studies and suggest, indirectly, that some of the ‘multifactorial’ effects mentioned above may have been absent for this study.

When analyzing all studies, including non-standard neurofeedback protocols, a non-significant effect on teacher ratings is found (also see: Cortese et al. 2016 for further overview). For example, training of the posterior alpha rhythm has failed to show clinical benefit in either hyperkinetic syndrome (Nall 1973) or epilepsy (Rockstroh et al. 1993). However, such findings could also be interpreted to suggest *specificity* in the EEG parameter trained for successful neurofeedback. In addition, while our conclusions are only generalizable to standard neurofeedback protocols, it is known that some clinical practices do not primarily focus on these standard protocols. Some clinical practices use ‘unconventional neurofeedback protocols’, such as the ones investigated by Van Dongen-Boomsma et al. (2013), as well as some more recently developed ‘modern protocols’ (e.g. Z-Score or LORETA Neurofeedback). However, a recent systematic review in this journal failed to find sufficient evidence for these non-standard approaches (Coben et al. 2018). Therefore, it is important that the clinical application of neurofeedback in clinics also more closely follows these recommendations and that neurofeedback organizations more formally recommend and educate this more strictly.

Whether neurofeedback is more or less cost-effective than medication and/or behavioural therapy is yet to be determined in sufficient detail. Broadly, though, neurofeedback usually requires 30–40 treatment sessions and, depending on geographical region, may cost between US $4000 and US $6000. In contrast, medication may be required for many years, possibly for 10 years or more. At an average cost of $2 per day, the overall cost of medication would amount to $3500 to $7000 for periods ranging between 5 and 10 years, to which would need to be added ongoing paedriatric and/or general practitioner service costs associated with the provision of prescriptions, medical check-ups and fractionalized costs of (severe) side-effects. The costs of multicomponent behaviour therapy, as it was used in the MTA study, due to its very intensive character including summer camps and parent trainings, is a more expensive treatment compared to neurofeedback (Arnold personal communication; August 9th 2018). Furthermore, the total economic burden—excluding treatment costs—has been estimated to be $ 15,036 per child with ADHD vs. $2848 per child without ADHD (Zhao et al. 2019), hence the lasting 32–47% remission rates reported imply a dramatic savings on economic burden independent of the treatment costs as well and posit neurofeedback as a cost-effective intervention, especially in regard to the long-term effects.

It is concluded, using a stricter version of the APA guidelines, that standard neurofeedback protocols in the treatment of ADHD can be considered as well-established and ‘efficacious and specific’, with medium to large effect sizes and 32–47% remission rates and sustained effects as assessed after 6–12 months.

## Disclosures

MA reports options from Brain Resource (Sydney, Australia); is unpaid research director of the non-profit Brainclinics Foundation, a minority shareholder in neuroCare Group (Munich, Germany), and a co-inventor on 4 patent applications (A61B5/0402; US2007/0299323, A1; WO2010/139361 A1; WO2017/099603 A1) related to EEG, neuromodulation and psychophysiology, but does not own these or receive any proceeds related to these patents; Research Institute Brainclinics received research funding from Brain Resource (Sydney, Australia) and neuroCare Group (Munich, Germany), and equipment support from Deymed, neuroConn and Magventure, however data analyses and writing of this manuscript were unconstrained. CRC reports a minor shareholding in Brain Resource (Sydney, Australia); MT reports being the Managing Director and co-owner of NeuroThrive, LLC. RD reports receiving research funding from NIMH (Grant No. R01MH100144), Commonwealth Health Research Board, Edwin Joseph Foundation, Riverside Foundation, and VuBay Foundation; is current President, Board of Directors, of the International Society for Neurofeedback and Research (ISNR).
